# Upper Respiratory Symptoms, Gut Health and Mucosal Immunity in Athletes

**DOI:** 10.1007/s40279-017-0846-4

**Published:** 2018-01-24

**Authors:** Candice Colbey, Amanda J. Cox, David B. Pyne, Ping Zhang, Allan W. Cripps, Nicholas P. West

**Affiliations:** 1 Menzies Health Institute Queensland and School of Medical Science, Griffith University, Griffith Health Gold Coast Campus, Southport, QLD 4222 Australia; 2 Faculty of Health, University of Canberra Research Institute for Sport and Exercise, University of Canberra, Canberra, ACT Australia; 3 Discipline of Physiology, Australian Institute of Sport, Canberra, ACT Australia

## Abstract

Upper respiratory symptoms remain the most common illness in athletes. Upper respiratory symptoms during heavy training and competition may impair performance. Preventing illness is the primary reason for the use of supplements, such as probiotics and prebiotics, for maintaining or promoting gut health and immune function. While exercise-induced perturbations in the immune system may increase susceptibility to illness and infection, growing evidence indicates that upper respiratory symptoms are related to a breakdown in the homeostatic regulation of the mucosal immune system of the airways. Balancing protection of the respiratory tract with normal physiological functioning requires dynamic orchestration between a wide array of immune parameters. The intestinal microbiota regulates extra-intestinal immunity via the common mucosal immune system and new evidence implicates the microbiota of the nose, mouth and respiratory tract in upper respiratory symptoms. Omics’ approaches now facilitate comprehensive profiling at the molecular and proteomic levels to reveal new pathways and molecules of immune regulation. New targets may provide for personalised nutritional and training interventions to maintain athlete health.

## Introduction

With the exception of injury, the most common medical presentation in elite athletes [1–3] is upper respiratory symptoms (URS). While the frequency of infectious URS in athletes is comparable to the general population, the timing does not follow typical seasonal fluctuations [4]. This pattern indicates that factors specific to the type of exercise and athlete behaviour can alter susceptibility to URS, particularly as episodes appear more frequently during periods of increased training load and around competition [5]. Successful competitive performance for an elite athlete is often determined by the narrowest of margins. Upper respiratory symptoms may have a range of detrimental effects on athletic performance, including reduced aerobic capacity, muscular strength, muscular co-ordination, speed of contraction, alertness and information processing [6–8].

**Fig. 1:**
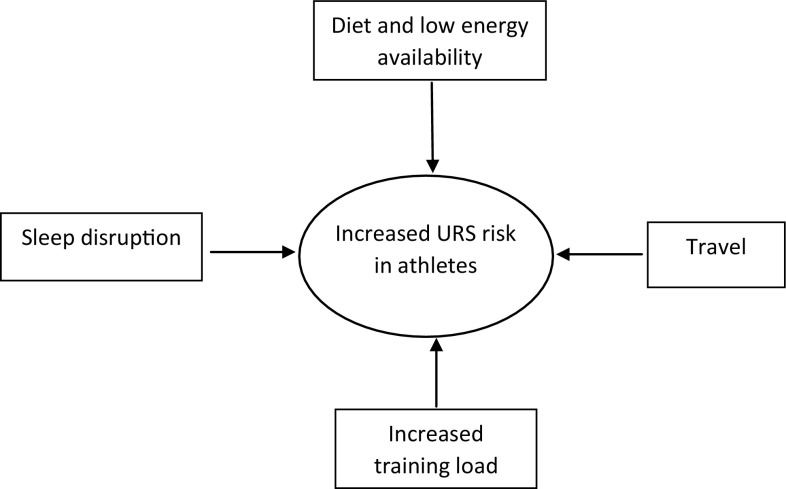
Factors contributing to upper respiratory symptoms (URS) in elite athletes include higher training load, sleep disruption, travel and jetlag and dietary alterations

While anecdotally the issue of URS is of high concern to athletes and coaches, few studies have directly quantified the effects of URS on performance outcomes. A study of elite swimmers indicated that mild illness had only trivial effects on the performance of female swimmers and a small harmful effect in male swimmers [9]. Mild URS did not impair submaximal and maximal performance in highly trained middle- and long-distance runners [10]. Many factors contribute to increased URS in elite athletes including travel, stress, low energy availability and poor sleep quality (Fig. 1) [11].

Acute and chronic exercise-induced perturbations of the mucosal immune system may be a factor in the patterns of URS in athletes. The mucosal immune system, including the airway epithelia and microbiota, is an integrated network of mechanical, cellular and humoural factors balanced to protect the host from environmental antigens while maintaining homeostasis. The importance of host microbiota in the regulation of the mucosal immune system has driven athlete interest in the use of nutritional strategies to maintain gut health. This review examines mucosal immunity, the microbiota and gut-respiratory axis and use of a systems biology approach in the context of URS and athlete health. We also consider the implications of this approach for personalised nutrition and intervention approaches to minimise URS in athletes.

## Upper Respiratory Symptoms in Athletes

Upper respiratory symptoms involve several non-specific symptoms of the upper airways including coughing, sneezing, congestion, sore throat, mucus production and bronchoconstriction [12]. Upper respiratory symptoms are thought to impair athletic performance, with athletes and coaches placing a high priority on preventing illness [13]. A recent study in elite athletes preparing for the Rio 2016 Olympics revealed a broad list of risk factors for URS, including sex, energy availability, stress, communal living and hygiene practices associated with increased URS [14]. Improved knowledge regarding these factors will enhance the monitoring, management and application of intervention strategies needed to maintain athlete health and maximise competitive sporting outcomes.

The frequency of URS is higher during prolonged intense training or in acute periods of increased training and competition [15]. Training history and fitness may provide a higher tolerance to high training loads and a lower risk of URS [16]. Epidemiological and observational evidence indicates immune perturbations associated with URS occur more strongly in response to endurance exercise, such as marathon running, swimming and triathlon, than in team sports [17]. Seminal research in the area of exercise and URS noted that marathon runners with higher training loads had an almost a two-fold greater risk of URS than with low training loads. Completion of a marathon led to a six-fold increase in the likelihood of URS than in runners who had trained but did not compete [18, 19]. Similar reports are also available in other sports. A 2-week intensified training period increased URS almost threefold in well-trained male cyclists [20]. A large body of evidence now supports the premise that prolonged periods of intense exercise training can increase susceptibility to URS.

## Aetiology of Upper Respiratory Symptoms

Infectious pathogens, in particular viruses, are considered the primary cause for URS in athletes. Interestingly though, a study of 32 elite triathletes, 31 recreational triathletes and 20 sedentary non-athletes, which monitored URS over 5 months, reported that out of 37 episodes of illness, only 30% were caused by an infectious agent [21]. Furthermore, a prospective 14-month analysis of URS in 70 elite athletes in several sports could not identify a bacterial or viral pathogen in 43% of episodes [22]. Cox et al. [22] demonstrated that in elite athletes only 57% of clinician-diagnosed upper respiratory infections were found to be of infectious aetiology. Although laboratory identification of pathogens has sample and methodological limitations [23], evidence from these moderately sized athletic cohorts is suggestive of URS having multiple aetiologies. One theory is that URS can be induced by increased exposure to aeroallergens that generate a hypersensitivity response [24]. Another possible explanation for URS is exercise-induced bronchoconstriction [25]. Exercise-induced bronchoconstriction is considered a consequence of airway drying related to hyperventilation that initiates an acute inflammatory response of the upper airways [26]. Exercise-induced bronchoconstriction is commonly reported in athletes with asthma and may relate to disease control [26, 27]. Despite differences in aetiology, whether infection, allergy or generalised inflammation, URS presents with similar signs and symptoms and has common effects on human performance. Given the uncertainty regarding the aetiology of respiratory symptoms in athletes, there are recommendations that symptoms be reported as URS instead of upper respiratory tract illness or infection (URTI) [28].

## Mucosal Immunity and Respiratory Illness

**Fig. 2:**
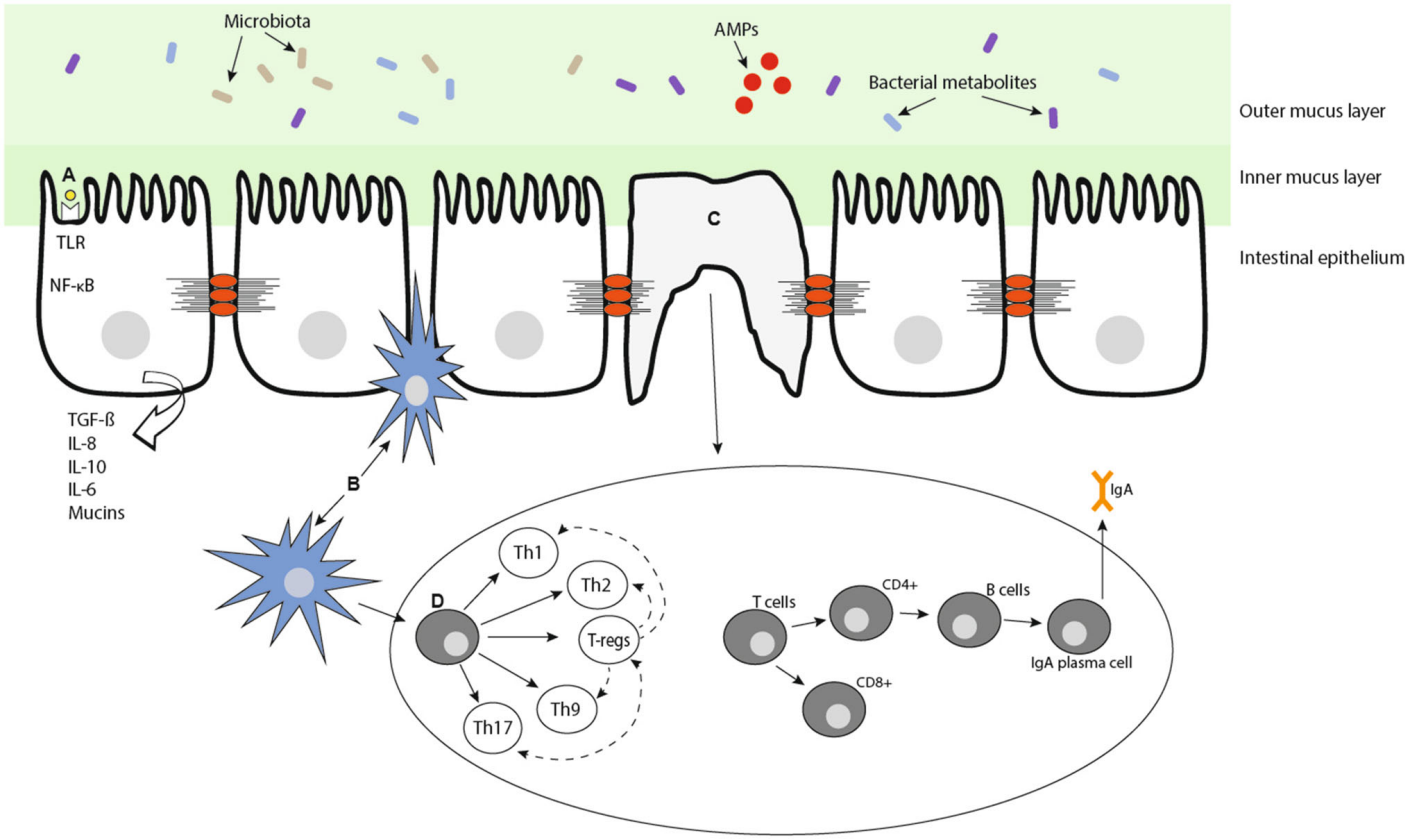
Schematic of the mucosal immune system. Interaction with environmental antigens **a** the microbiota, microbial metabolites, antimicrobial proteins (AMPs) (**c**) and dendritic processes **b** provide the mucosal immune system with multiple transient activation signals. Antigen invasion is prevented by the mucus layer, its constituent components and ciliated airway cells. T- and B-cell subsets **d** provide multiple, but highly plastic cell differentiation programmes. *CD* cluster of differentiation cells, *Th* T-helper, *IgA* immunoglobulin A, *Il* interleukin, *NF*-*kB* nuclear factor-kappa B, *TGF*-*β* transforming growth factor beta, *TLR* toll-like receptor, *T*-*regs* regulatory T cells

The clinical need for a differential diagnosis of URS in athletes has led to an increasing focus on the mechanisms of mucosal homeostasis in the respiratory tract. The mucosal epithelial/immune system, referred to hereon as the mucosal immune system, lines the external surfaces of the body. As the interface with the environment, the mucosal immune system orchestrates a delicate balance between protection of the body from external antigens and maintenance of homeostasis for normal physiological functioning [29]. To maintain this balance, the mucosal immune system has specialised effector and regulatory mechanisms to neutralise, remove and promote tolerance to antigens without inducing an inflammatory response (Fig. 2). While not separate from the systemic immune system, the localised defence factors and regional regulation of inflammatory processes at the mucosa [30] mean the mucosal immune system is often considered independent from other immune processes in the body.

A breakdown in several mechanisms of the airways may be involved in URS. The airway epithelia of the nose and throat are constantly exposed to external antigens and consist of several cell types that each play a different role in facilitating protection of the respiratory tract [31]. The airway epithelium regulates the secretion of saliva and composition of its constituent components, including mucus, secretory immunoglobulin A (SIgA), and humoural innate immune proteins, such as lactoferrin, lysozyme, and the defensins. Together with the epithelium, saliva and its various constituents form a physical and chemical barrier to prevent chronic inflammatory processes occurring in response to the constant exposure of antigenic material passing through the respiratory tract. The airway epithelium, in combination with localised antigen-presenting cells, links the innate and adaptive immune system via production of cytokines and chemokines to initiate inflammation should infection occur. However, the respiratory tract contains specialised lymphoid cells and immune mechanisms to exert an immunosuppressive influence on adaptive immune processes to tightly control inflammatory responses.

Important differences between airway and systemic immune compartments are being identified that provide insights into the aetiology of airway illness. A recent analysis of cluster of differentiation (CD)4^+^ T cell subsets in the human upper airway mucosa under non-inflammatory conditions reported that regulatory T-cells (T-regs) secreted substantially higher quantities of interleukin (IL)-10 than T-regs in peripheral blood [32]. Interleukin-10 is an immunoregulatory cytokine that exerts a suppressive influence on inflammatory responses and is central to resolving inflammation. Interestingly, this study also observed that inducible T-regs (foxp3^+^helios^−^) in the airways contained the highest frequency of IL-17-producing cells of the CD4^+^ T-cell subsets and that a higher percentage of foxp3^−^CD4^+^ T cells produced IL-10 than peripheral blood [32]. The higher frequency of inducible T-regs producing IL-17 may be important for the transport of SIgA through the induction of T-helper (Th)-17 cells required for T-cell-dependent immunoglobulin A production [33]. In recent years, recognition of the role of innate lymphoid cells as regulators of the mucosal immune system has been confirmed [34]. Immunological processes in the airway mucosal immune system exert an immunosuppressive influence to maintain homeostasis. A better understanding of these specific processes, particularly under exercising conditions, is required if strategies are to be developed to limit URS in athletes.

Dysfunction of the mucosal immune system is associated with increased illness, including URS. The lack of infectious sequelae in the URS of athletic groups has led to interest in whether dysregulated inflammatory processes underpin the patterns of illness observed [4]. Hypersensitivity reactions, such as asthma and allergy, are increasing in prevalence in the general population. Undiagnosed allergy and asthma are recognised as an additional cause of unexplained URS in athletes and the need for appropriate diagnosis and management is deemed a priority [4]. However, factors specific to elite athletes and sports modalities may also induce transient, non-allergic, asthma-like airway inflammation. Damage to the airway lining from hyperventilation via breathing through the mouth or from unfavourable conditions (cold or polluted air) is recognised as a cause of airway problems in athletes [35].

Evidence from animal research suggests that epithelial damage-related chemokines may mediate non-allergic asthma-like inflammation in the airways through type 2 innate lymphoid cells (ILC2 s) in the absence of adaptive Th-2-driven immunity [36]. Type 2 innate lymphoid cells are immune cells phenotypically similar to Th-2 lymphocytes that lack antigen receptors and secrete IL-4, IL-5, IL-9 and IL-13. Type 2 innate lymphoid cells respond to airway epithelial-derived cytokines, such as IL-25, IL-33 and thymic stromal lymphopoietin [37], to initiate a type 2 inflammatory event (asthma like) in the respiratory tract. Activation of ILC2s in the respiratory tract has been linked with allergic and non-allergic asthma-like airway inflammation in animal models. In rag2^−/−^IL2^−/−^ mice that lack lymphocytes (including ILC2s), administration of epithelium-derived IL-33 via inhalation does not induce asthma-like inflammation. However, an asthma-like response can be partially induced by engraftment of ILC2s in the respiratory epithelia [36]. Evidence that ILC2s mediate non-allergic asthma-like symptoms in response to airway epithelial damage provides a mechanism that may explain the idiopathic symptomatology being described as URS in athletes.

In general, athlete-related URS are considered to be local to immune mechanisms in the respiratory tract rather than from exercise-induced systemic inflammatory processes, which has implications for using systemic markers as a surrogate measure of URS risk. While studies have examined numerous systemic and mucosal cellular [38] and humoural [39, 40] immune parameters in the context of risk of URS in athletes, only SIgA has shown moderate diagnostic value [41], although its utility is limited given large intra- and inter-individual variation [42]. The role of other humoural factors in saliva is also becoming apparent with micro-proteomic technology. The application of microproteomic technology using the saliva of healthy individuals identified over 1000 secreted proteins, while a comparison of the proteins in saliva between patients with influenza and healthy controls identified 162 differentially expressed proteins associated with the respiratory mucosal immune response [43]. Research in non-athletic cohorts has also failed to identify systemic inflammatory markers with strong diagnostic value for respiratory infection, even within cohorts admitted to hospital [44]. Animal and human research provides some evidence that inflammatory cytokines associated with excess body mass, such as IL-1β, may exacerbate ILC2 and ILC3 airway responses [45], indicating that systemically released serum cytokines could modulate airway responses to harmful and innocuous stimuli. Whether this process relates only to secretions from adipose tissue and not muscle-derived cytokines from exercise is yet to be established.

Biological factors may also limit the utility of using systemic markers as a surrogate for mucosal immune processes that underpin URS [46]. Mucosal compartments contain site-specific immune cells that govern cell receptor repertoires and functions. Exercise is recognised to induce changes in the frequencies of systemic cell populations, in particular T-regs [47, 48]. These changes in the frequency of systemic cell populations are linked to the beneficial anti-inflammatory effects of exercise but could mediate the increased susceptibility to URS in some athletes [49, 50]. Interestingly, investigations in patients with inflammatory bowel disease found no correlation between changes in the percentage of peripheral CD4^+^CD25highFOXP3^+^ T-regs and intestinal FOXP3+ T-regs with clinical activity [51], suggesting that changes in the frequency of T-regs in blood may be independent of those in the mucosa.

Furthermore, the continued identification in the mucosal immune system of new rare lymphocyte subsets and subsets that co-express lineage-specific transcription factors and chemokine receptors is re-defining the traditional view of adaptive immunity as being either a Th-1- or Th-2-driven response [52]. There appears to be far greater phenotypic plasticity amongst CD4^+^ T-cell subsets, greater overlap between subsets than previously recognised, and striking differences between sites in cell composition and function, particularly in relation to the airway [53, 54]. Whether immune cells in peripheral blood reflect the frequency and function of immune cells in the airways is yet to be established. Taking account of the complexity of the mucosal immune network will be necessary before changes in susceptibility to URS can be determined via peripheral markers.

## Microbiota, Nutrition Supplements and Upper Respiratory Symptoms

Interest in the microbiome has grown in recent decades with strong evidence that host microbes are essential for the ontogeny of the immune system and play a central role in health and disease. To date, the major focus has been on gut bacteria, now estimated to number ~ 10^14^ colony-forming units across 500 species. The intestinal microbiota is involved in the vital functions of food digestion, nutrient production, priming of the immune system, protection from ingested pathogens and production of short-chain fatty acids [55, 56]. Host microbes are proposed to exist in a continuum from symbiosis in healthy individuals to dysbiosis in disease. Basic and human research has implicated the intestinal microbiota in intestinal diseases, such as inflammatory bowel disease and colon cancer, along with extra-intestinal diseases including obesity and metabolic syndrome [57]. The profound influence of the microbiota in health and disease has led to the funding of large-scale collaborative and international consortia [58, 59] to catalogue an ecosystem that is now considered an organ system in its own right.

The intestinal microbiota is able to alter homeostasis at distant mucosal sites, including the respiratory tract, through the common mucosal immune system [60]. Existence of the common mucosal immune system has long been accepted through evidence that vaccination elicits protection at (mucosal) sites distal to the initial mucosal site of immunisation [61]. A key mechanism of microbial influence on distal mucosal sites from the intestine is through induction of immunoglobulin A-producing plasma cells [62]. Luminal sampling of host microbiota by dendritic cells in the intestinal mucosa is considered a key aspect of host-microbe signalling [63]. Antigen-primed B cells migrate via the thoracic duct throughout the mucosa and differentiate into plasma cells. At these sites, plasma cells produce SIgA that is transported to mucosal surfaces to act as a primary molecule in immune exclusion of environmental antigens [64]. Animal and human studies demonstrate the production of antigen-specific SIgA occurs in the intestine and respiratory tract in parallel [65]. The intestinal microbiota is also implicated in the maintenance and differentiation of T-cell subsets located in distal mucosal sites [66]. Animal research highlights that specific clusters of Clostridia promote the accumulation of T-regs in the intestine [67]. More recently, the microbiota has also been found to promote T-regs that express the Th-17 phenotype that, in conjunction with T-regs, regulate homeostasis in the mucosa [68]. There is strong evidence that the intestinal microbiota plays a key role in programming of the mucosal immune system.

There is strong interest in gut health and the host microbiota for athlete health yet a paucity of experimental research in athletes. A recent study reported that regular moderate physical activity improved anxiety, SIgA and total culturable oral bacteria counts in 19 female athletes [69]. While this outcome suggests a positive effect of moderate exercise on oral microbial diversity, no mention is made of dietary profiling of the athletes, and molecular methods would provide a more detailed understanding of bacterial diversity given many species cannot be cultured. Only one study has examined intestinal bacteria in elite athletes, a comparison between a professional international-level rugby team (*n* = 40) with a non-rugby-playing, healthy, low body mass index (BMI) (22 ± 1.8 kg/m^2^; mean ± standard deviation) and a healthy high BMI (31.2 ± 3.0 kg/m^2^) cohort. The inclusion of a healthy high BMI group provided a direct comparison of the microbiota with BMI, which has been shown to have lower diversity in individuals with high BMI [70]. The study found a higher microbial diversity in the rugby players that appeared to relate to the dietary intake of the athletes, with the athletes consuming more total calories and higher amounts of all macronutrients [71].

The outcomes in the rugby players are consistent with work in our laboratory, with national-level triathletes having a greater diversity of intestinal microbiota than non-athletic healthy individuals (unpublished data). Diet has a profound effect on the intestinal microbiota [72] and is proposed to be the primary reason that the microbiota may be more diverse in athletes [73]. However, a recent study in 39 healthy active adults controlled for age, BMI and diet found that cardiorespiratory fitness was positively correlated with the diversity of the gut microbiota [74]. This has important implications for exercise prescription and potentially for decisions by athletes on a sport-by-sport basis for the use of gut health supplements. As yet, there is little information on whether athletes who are more susceptible to URS differ in the diversity of intestinal or oral microbiota compared to healthy athletes.

The influence of the intestinal microbiota on respiratory health underpins the interest by athletes in the use of gut health products, such as probiotics and prebiotics, to reduce susceptibility to URS. Evidence on the effectiveness of probiotics in athletic groups is mixed (Table 1), with some studies showing a reduction in the rate of URS while other studies report a reduction in the duration or severity of illness but no effect on incidence [75, 76]. Differences in the effectiveness of probiotic supplements relate to the type of sport (endurance vs. team sport), the training history of the athlete, the training load being undertaken along with supplement-specific differences in the strain(s), method of delivery and duration of supplementation [77].

**Table 1:** Effect of probiotic supplementation on upper respiratory symptoms (URS) in athletic cohorts ranging from healthy active individuals through to elite athletes

References	Study design and participants	Intervention	Impact on URS
Clancy et al. [119]	Double-blind placebo-controlled trial of 18 healthy and nine fatigued recreational athletes over 4 weeks	Probiotic (*Lactobacillus acidophilus* LAFT1-L10 strain) daily	Reversal of defect in IFN-γ secretion from T cells (viral control mechanism)
Cox et al. [120]	Double-blind placebo-controlled trial of 20 healthy, elite male distance runners over 16 weeks	Probiotic (*Lactobacillus fermentum* VRI-003 strain) daily	Reduced incidence of URS by 50% and reduced severity of symptoms and trend for higher IFN-γ secretion from T cells (*p* = 0.07)
Gleeson et al. [79]	Double-blind placebo-controlled trial of 84 endurance athletes over 16 weeks	Probiotic (*Lactobacillus casei* Shirota strain) daily	Reduced the number of URS episodes by ~ 50%; higher SIgA level in those taking probiotics
Haywood et al. [86]	Single-blind, placebo-controlled, double-arm crossover trial of 30 rugby players, 4 weeks per treatment separated by a 4-week washout	Probiotic (*Lactobacillus gasseri, Bifidobacterium longum, Bifidobacterium bifidum* strains) daily	No difference in the incidence of URS
West et al. [87]	Double-blind placebo-controlled trial of 88 well-trained recreational cyclists over 11 weeks	Probiotic (*Lactobacillus fermentum* VRI-003 strain) daily	No significant effects on URS; reduction of LRI in male cyclists by a factor of 0.31 but a 2.2-fold increase in LRI in female cyclists
Gleeson et al. [76]	Double-blind placebo-controlled trial of 54 endurance athletes over 16 weeks	Probiotic (*Lactobacillus salivarius* strain) daily	No difference in the incidence of URS
Kekkonen et al. [75]	Double-blind placebo-controlled trial of 141 marathon runners over 3 months	Probiotic (*Lactobacillus rhamnosus* GG strain) daily	No difference in the incidence of URS
West et al. [88]	Double-blind placebo-controlled trial of 465 physically active individuals for 150 days	Probiotics (*Bifidobacterium animalis* subsp. *lactis* Bl-04) daily or *Lactobacillus acidophilus* NCFM and *Bifidobacterium animalis* subsp*. lactis* Bi-07 daily	Bl-04 associated with a significant 27% reduction in the risk of URS compared with placebo

*IFN* interferon, *LRI* lower respiratory illness, *SIgA* salivary immunoglobulin A

Research in our group has also observed sex differences in the effect of probiotic supplements on URS. In this study of 99 competitive cyclists (64 male and 35 female individuals; age 35 ± 9 and 36 ± 9 years), respiratory symptoms were lower by a factor of 0.31 [99% confidence interval (CI) 0.07–0.96] in male individuals but increased by a factor of 2.2 (99% CI 0.41–27) in female individuals during 11 weeks of supplementation with *Lactobacillus fermentum* (PCC^®^) [78]. The mechanisms underpinning the beneficial effects of probiotic supplements on URS may include modulation of serum cytokines and SIgA, and changes in the percentage and functional capability of innate and adaptive immune cells [76, 79]. Animal and in vitro studies also show that probiotic strains can increase the expression of mucin genes and mucin secretion from intestinal epithelial cells along with secretion of antimicrobial peptides, which would enhance the barrier function of the mucosa [80]. At this stage, various immune mechanisms may explain the beneficial effects of probiotics on URS in athletes.

Few studies have examined the effects of prebiotics on URS and the immune system in athletes. The effects of β-glucan, a long-chain non-digestible carbohydrate, on URS and the immune system have been examined in acute exercise extended training (10–90 days) with mixed outcomes. The administration of β-glucan for 28 days in 182 healthy adults before a marathon was associated with a significant 37% reduction in the number of URS symptom days compared with placebo [81]. This study also reported that 10 days of β-glucan supplementation prior to a 60-min cycling session in a hot (45 °C) and humid (~ 50% humidity) environment was associated with a 32% increase in SIgA 2 h post-exercise in 60 healthy active individuals [81]. A longer duration supplement period of 90 days in 50 athletes in a placebo controlled trial also reduced the incidence of URTI and increased the number of circulating natural killer cells [82].

In contrast to these studies, consumption of β-glucan for 2 weeks before, during and 1 day after 3 days of exercise in which athletes cycled for 3 h per day at 57% of their maximal wattage had no significant effect on URTI in the 2 weeks post-exercise [83]. Research by our group examining 28 days of butyrylated high-amylose maize starch supplementation in 41 recreational cyclists observed significant increases in faecal short chain fatty acids, the abundance of faecal *Parabacteroides distasonis* and *Faecalibacterium prausnitzii*, and in the concentration of plasma IL-10 and tumour necrosis factor-α [84]. *Faecalibacterium prausnitzii* is recognised for a range of important mucosal and systemic anti-inflammatory effects and for promoting gut health [85]. Beneficial effects of prebiotics and probiotics have been shown for a variety of sports, including team sports [86], highly active elite triathletes along with recreational endurance athletes [76, 79, 87], and healthy active individuals undertaking general exercise, team sports and endurance sports [88].

The use of colostrum for gut health and immune function has also been extensively studied in athletes. Colostrum is rich in antibodies and growth factors, which may be protective by modulating the gut microbiota and improving intestinal permeability. A focus of research using colostrum supplementation has been to prevent heat-related exercise-induced intestinal permeability albeit with mixed results [89, 90]. In one study, supplementation with bovine colostrum during 8 weeks of endurance running training increased intestinal permeability compared with whey protein powder [91]. A recent meta-analysis on the use of bovine colostrum for URS during exercise indicated that supplementation reduced the incidence rate of URS days (rate ratio 0.56, 95% CI 0.43–0.72) and URS episodes (0.62, 95% CI 0.40–0.99) [92]. While evidence suggests limited effects on gut and immune markers [93], the reduction in URS highlights the potential for colostrum forming one part of a strategy to maintain health in athletes.

More recently, the contribution of the oral microbiome in respiratory illness has generated significant interest. Overall, there is considerable diversity within the oral microbiome [94]. A comparison of the microbe-specific peptide composition of saliva quantified more than 2000 microbial proteins from 50 bacteria genera, with significant differences in the proteins and species between individuals and in response to food consumption and cleaning teeth [95]. Differences in the composition of the oral microbiome have also been observed in individuals with severe asthma, who had higher numbers of microbes associated with eosinophilia compared with healthy controls and individuals without severe asthma [96]. Interestingly, a reduction in the diversity of the oral microbiome has been observed in oral and respiratory disease, which is similar to observations regarding the intestinal microbiota and intestinal disease [97]. Whether this is causal is yet to be determined. A comparison in 28 healthy individuals of the microbiome of the upper airways also revealed significant differences in the composition of the nasal and oral microbiome but a significant overlap between the microbe composition of the oral cavity and the lungs [98]. The existence of a diverse and compositionally different microbiota in the upper airways along with interactions with the intestinal microbiome suggests a more detailed characterisation of mucosal immune homeostasis may be necessary to understand the pathogenesis of URS in athletes.

## Integrated Immune-Microbial Biomarker Profiling: A Systems Approach

Athlete-related URS appears to be a heterogeneous condition with a complex pathophysiology encompassing an interaction between the immune system, microbial elements and environmental factors. Traditionally, immune profiling in exercise immunology and across other disciplines has focused on the analysis of single or a limited set of immunological parameters. The mucosal immune system includes a wide array of cells, humoural factors and mechanical barriers, the nature of its activation is multi-factorial and transient, and its constituents share overlapping functions that may be redundant, antagonistic or synergistic within regulatory cascades (Fig. 2). While immune phenotype is traditionally measured at the proteomic level, the plasticity and adaptability of immune activity is governed by molecular transcriptional and translational regulation and post-translational modifications [99, 100]. In many cases, the correlation between molecular immune activity and protein abundance is poor, indicating that the dynamic nature of immune regulation is not linear and, in many cases, uncoupled with the measured phenotype [101]. The complexity of the immune system working across several levels therefore requires the integration of molecular, phenotypic and behavioural data to identify regulatory networks that govern homeostasis and identify critical molecules driving aberrant inflammatory activity [102].

The use of ‘omics’ technology to evaluate immune status in a holistic manner, including metabolites (metabolomics), proteins (proteomics), messenger RNA (transcriptomics) or genes (genomics), and metagenomics (microbiota) [103] is providing unprecedented insight into the ‘immunopathology’ of disease. New technologies, such as the nCounter^®^ Analysis System (NanoString Technologies, Inc., Seattle, WA, USA) that utilise small sample volumes provide the opportunity to undertake simultaneous immune genome and phenotype analyses. Mass cytometry for immune cell phenotyping overcomes the limitations of flow cytometry spectral overlap that restricts routine cell phenotyping to 8–12 immune markers. Mass cytometry allows for the simultaneous quantitation of 30–40 cell markers, which provides the opportunity to interrogate multiple cell types and signalling pathways for a more holistic understanding of biological function [104]. The ability to simultaneously characterise genomic and proteomic factors is revealing biomarkers that are being used for diagnostic purposes and the stratification of patients into disease risk groups [105, 106]. In the sports medicine setting, the use of genomic and proteomic analyses would extend traditional cell phenotyping to include functional assessment and provide important information on changes in immune capability that may underpin URS.

**Fig. 3:**
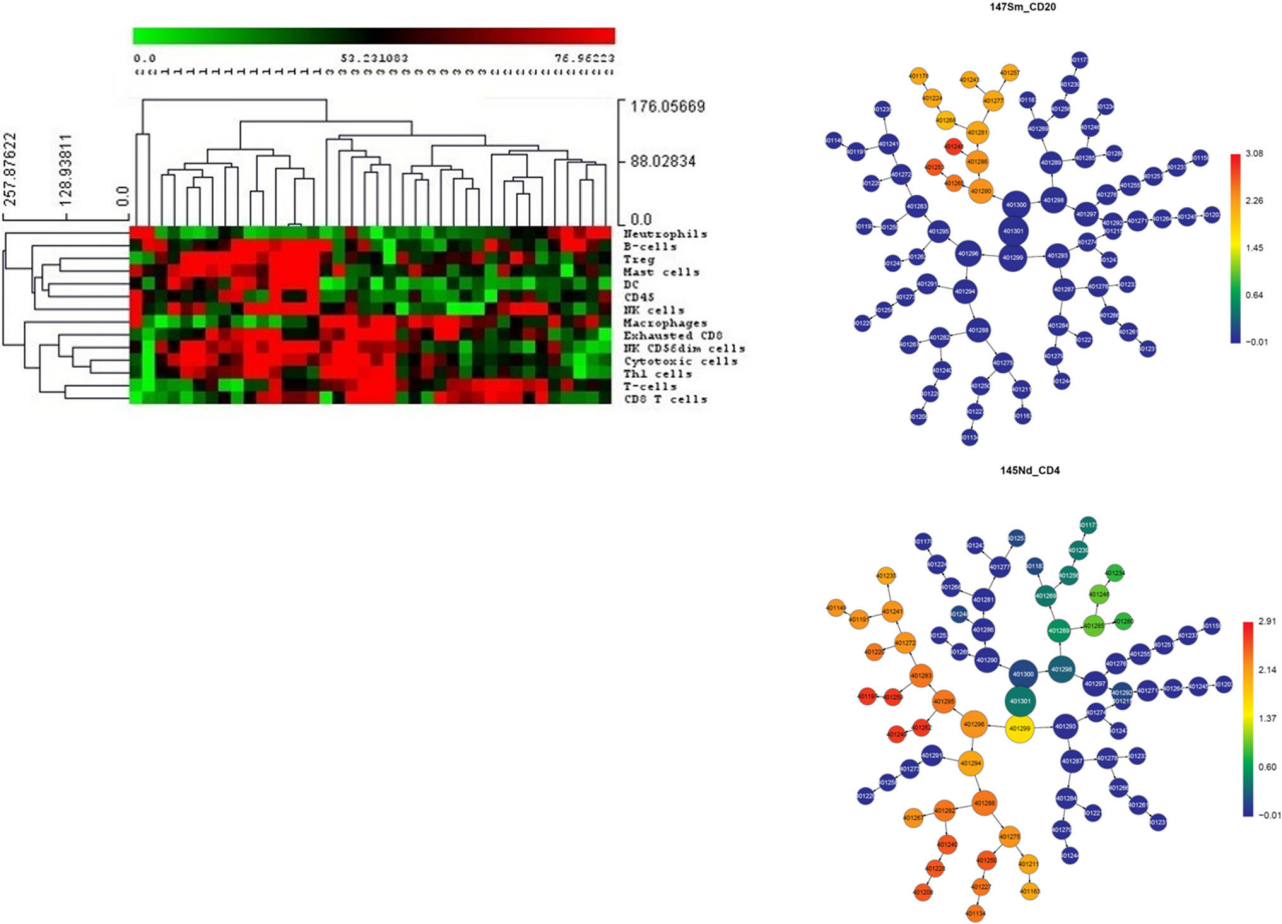
Analysing large data sets. Multi-parameter data sets require data visualisation and data reduction techniques to identify patterns between analytes of interest and that separate study groups under investigation. Cluster algorithms (left-hand figure) order cell types (rows) through NanoString immune gene expression by individuals (columns) to reveal shared and distinct patterns. In this case, the groups represent obese (1), endurance (2) and team sport (3) groups. *Red* is high expression and *green* is low expression. CITRUS (Cluster identification, characterisation and Regression; Cytobank, Santa Clara, CA, USA) (right-hand figures) for mass cytometry groups cells into nodes based on similarity of marker expression to reveal distinct patterns of cells/receptors between groups. Joined nodes represent phenotypic similarity and lineage relationships. In this diagram, the difference in expression of CD20 and CD4 between athletic groups is depicted. Red is high expression and *blue* is low expression. *DC*, *NK* natural killer, *Th* T-helper, *Treg* regulatory T cell

The generation of large biological datasets is recognised to pose substantial challenges in relation to data analysis and interpretation. Integration of large data sets from high-throughput technology requires the use of systems biology and machine learning approaches (Fig. 3). These approaches aim to build models of biological systems that incorporate interactions between genome, metagenome and the environment. Systems biology has shown promise in cancer, vaccine development, gastroenterology and in understanding inflammation in ageing [102, 107–109]. Our group has used these approaches to highlight the utility of using intestinal permeability measures as a tool for predicting type 2 diabetes mellitus risk [110]. Furthermore, the use of omics biomarker profiling is showing value for the identification of predictive markers in cancer settings [111]. Sports medicine is likely to be one of the next frontiers to move beyond the conventional focus of examining discrete targets for diagnostic and therapeutic purposes and embrace systems approaches for athlete health and performance. Integration of sequencing and high-throughput technologies using computational biology modelling approaches may provide important information on how perturbations in immune, microbial or environmental factors can lead to URS in athletes.

## Future Directions

In recent years, the advancement, affordability and availability of high-throughput analytical methods has increased substantially. High-throughput methods are being applied to a broad range of research questions to reveal highly detailed information about the complex biological interactions underpinning disease [112, 113]. These developments are facilitating the search for biomarkers used to monitor, predict and identify disease and pathophysiological processes. As a discipline, there is a long history in exercise immunology of examining the effects of exercise on salivary proteins given the ease of saliva collection [114, 115]. In-vivo studies utilising micro-proteomics technology for salivary assessment between athletes that experience URS compared with those who do not experience URS may reveal key molecules that alter susceptibility to URS. Furthermore, the use of micro-proteomics in nasal secretions would also offer new strategies to understand URS in athletes. Traditionally, large secretion volumes are required from healthy individuals for analysis of nasal proteins, which would be overcome through the use of micro-proteomics. Identification of new immune proteins in secretions of the airways would provide biomarker targets for assessment in exercise-induced suppression and URS.

The airway microbiome (nose, mouth and respiratory tract) represents an intriguing research area for exercise immunology. Given the various dietary practices of endurance vs. team sport athletes, and weight-restricted athletes, there is a unique opportunity to examine the role of dietary practices and exercise on the composition of both the intestinal and airway microbiome. Follow-up studies are then needed to examine whether changes in the composition modify mucosal immune control and susceptibility to URS. Furthermore, whether modification of the airway microbiome might reduce susceptibility to URS in the same manner that faecal transplants have altered disease outcomes in the colon is a tantalising prospect. Early-phase studies for the use of topical nasal sprays for URTI by modifying the nasal microbiota are well tolerated and safe in healthy adults [116], suggesting that therapies targeting the airway microbiota for URS are under consideration. While not yet conclusive, the consumption of probiotics has shown efficacy for URS and gastrointestinal illness in athletes. Intriguingly, there are few studies characterising the intestinal microbiota in athletes [73]. Research in gut microbiota is moving beyond just characterisation of phyla towards an understanding of structure function for targeted manipulation to elicit specific health benefits [117]. Future work to examine the effect of acute and chronic exercise on microbes of the gut might identify exercise- or sport-specific alterations in individual bacterial phyla, such as butyrate producers, that permit implementation of personalised probiotic regimens.

Deciphering aberrant processes in the mucosal immune system that denote dysfunction or altered susceptibility to infection requires a schema for the intricate balance between airway homeostasis and inflammation. Omics technology and use of approaches such as systems biology to integrate and construct multi-dimensional regulatory networks may shed light on the immunopathology underpinning URS. There is a need to undertake simultaneous molecular and protein phenotyping from mucosal and systemic samples to gain a better insight into immune status and to determine the relevance of sampling from one site for insight into the status of another. This type of work needs to examine whether rare immune cell populations co-exist and whether they exhibit similar functional capacity [118]. Such approaches may identify combinations of biomarkers that more accurately allow for earlier diagnosis of dysregulation at mucosal surfaces or increased susceptibility to URS. Within the context of elite athletes, biomarkers associated with URS could be used within the clinic to flag at-risk athletes who may benefit from closer assessment and intervention. The biomarkers could also be used to monitor athletes’ responses to training, dietary or therapeutics intervention and to optimise illness treatment.

## Conclusions

Upper respiratory symptoms may have a negative impact on athletes in heavy training or during competition by altering psychological and/or physiological capacity. The differential diagnoses of URS in athletic settings imply that mucosal immune dysregulation may underpin a substantial number of cases of URS. Maintenance of homeostasis in the respiratory tract involves complex immune, microbial and environmental interaction. Supplements that modify gut bacteria, including probiotics and prebiotics, are popular with athletes at all levels and a better understanding of the effects of exercise on the microbiota may lead to more personalised and effective supplement regimes. Identifying the role of the oral microbiome in URS and whether these microbes can be manipulated is a promising area of research. Use of new technology and data approaches in studies combined with clinical outcomes will provide a better understanding of the relationship between exercise and the mucosal immune system in URS. Improved understanding of this relationship will lead to nutrition and training strategies to improve gut and immune function and reduce the impact of URS on athletes.
